# Supplementary material for: Regulation of vacuole fusion in stomata by dephosphorylation of the HOPS subunit VPS39
Source: Plant J. 2025 Dec 19;124(6):e70640. doi: 10.1111/tpj.70640 (PMC12716901; doi:10.1111/tpj.70640)
Supplement: Supplementary file 1 — Figure S1. Stomata from epidermal fragments used for phosphoproteome. Bright‐field image of epidermal fragments after treatment in opening or closing conditions. Figure S2. Chimera X protein folding alignments of VPS39. (a) AlphaFold predictions and alignment of VPS39 orthologs from Marchantia polymorpha, Physcomitrium patens, Oryza sativa, and Arabidopsis thaliana. All plant VPS39 structures are predicted to have two large unstructured loops similar to Arabidopsis. (b, c) Neither Sacharomyces cerevisiae or human VPS39 proteins show similar unstructured domains as Arabidopsis. Data S1. Confidence intervals for bootstrap analysis from generalized linear mixed models for stomata assays. Data S2. Proteome and phosphoproteome data for proteins discussed in this study. Table S1. Comparison of medians of aborted embryos from Figure 3B. Table S2. Pairwise comparison of medians for unfertilized ovules from Figure 3C. Table S3. Reference phosphopeptides for PHOT1 and OST1. Table S4. HOPS, CORVET, and vacuolar SNARE proteins detected in proteome. Table S5. Detected phosphorylation of HOPS, CORVET, and vacuolar SNARE proteins. Table S6. Primers used in this study. [file TPJ-124-0-s001.zip › tpj70640-sup-0002-supinfo.pdf]

SUPPLEMENTAL INFORMATION

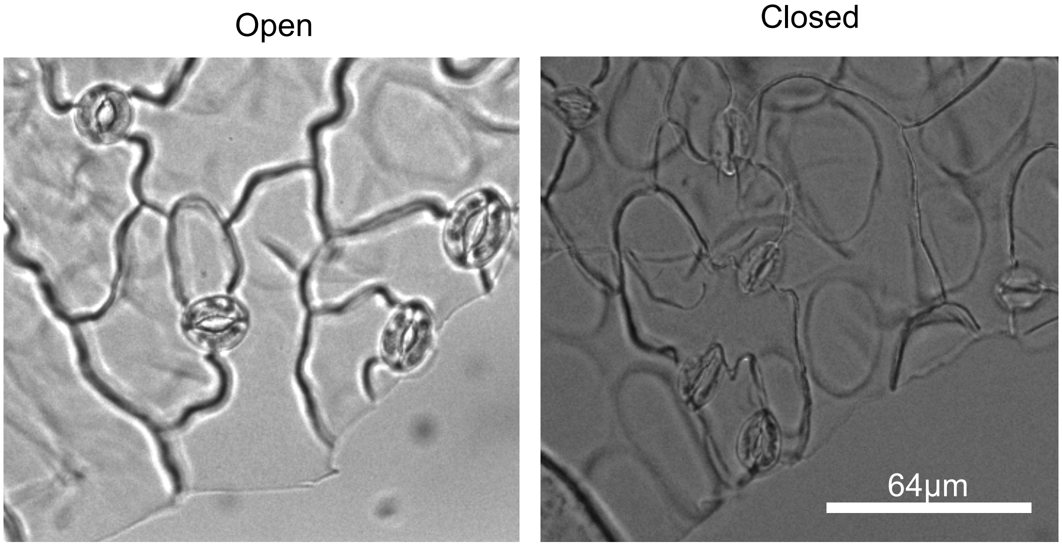

Supplemental Figure 1. Bright-field image of epidermal fragments used in proteome and phosphoproteome. Bright-field image of epidermal fragments after treatment in opening or closing conditions as used for phosphoproteomics.

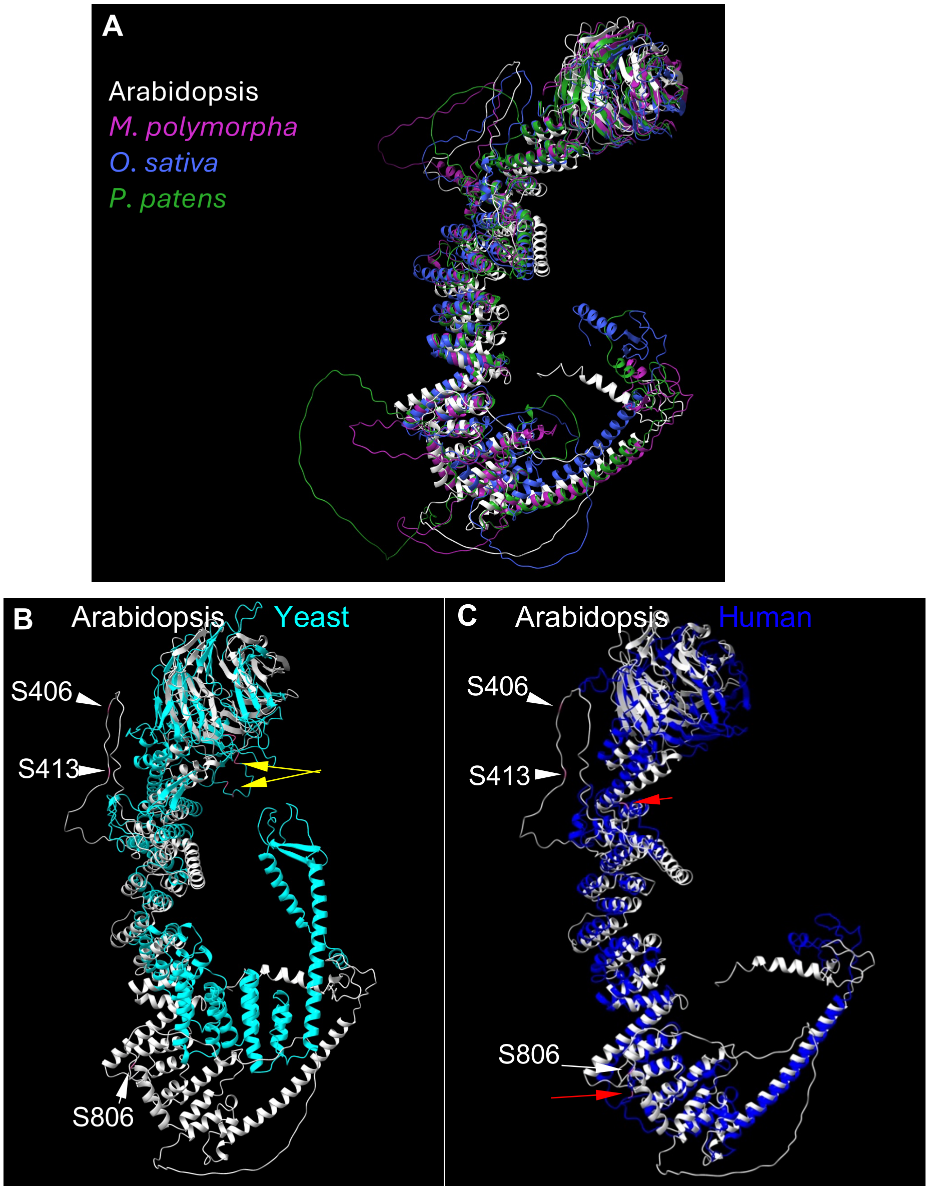

Supplemental Figure 2. Chimera X protein folding alignments of VPS39. (A) AlphaFold predictions and alignment of VPS39 homologs from *Marchantia polymorpha* (magenta), *Physcomitrium patens* (green), *Oryza sativa* (blue) and *Arabidopsis thaliana* (gray). All plant VPS39 structures are predicted to have two large unstructured loops similar to Arabidopsis. (B-C) Neither *Sacharomyces cerevisiae* (B) or human (C) VPS39 proteins show similar unstructured domains as Arabidopsis. (B) Arrowheads show position of S406, S413, S806 in Arabidopsis and yellow arrows point to phosphorylated residues in the yeast protein (S246, S247, S249, and S250). C) Red arrow points to S441 and T646 in human VPS39, which have been detected as phosphorylated.

Supplemental Dataset 1. Details on generalized linear mixed models for stomata assays

Confidence intervals for bootstrap analysis from Generalized linear mixed models for stomata assay, WT vs *vps39-2* (Fig 1F)

Model containing genotype was favored with a p-value of 0.01.

Biological replicates (n)	# Guard cells	#plants
WT	97	5
vps39-2	139	8

Comparison	95% confidence interval for bootstrap analysis
WT - *vps39-2*	[0.31, 9.66]

Confidence intervals for bootstrap analysis from Generalized linear mixed models for stomata assay, WT vs *vps39-2* vs, *vps39-2* VPS39^{SD}-GFP vs *vps39-2* VPS39^{SA}-GFP (Fig 3E)

Biological replicates (n)	# Guard cells	#plants
WT	408	23
vps39-2	436	26
vps39-2 VPS39^{SA}-GFP	107	9
vps39-2 VPS39^{SD}-GFP	97	9
vps39-2 VPS39-RFP	214	9

Model containing genotype was favored with a p-value of 1.304×10^{-7}. Data from the first stomata experiment was added for the WT and *vps39-2* genotypes to improve the robustness of the model.

Comparison	95% confidence interval for bootstrap analysis
vps39-2-WT(*)	[-3.4, -0.93];
vps39-2 VPS39^{SA}-GFP – WT (*)	[-2.12, -0.11]
vps39-2 VPS39^{SD}-GFP – WT (*)	[-2.13, -0.09]
vps39-2 - *vps39-2* VPS39^{SA}-GFP (NS)	[-2.27, 0.25]
vps39-2 - *vps39-2* VPS39^{SD}-GFP (NS)	[-2.31, 0.14]
vps39-2 VPS39^{SA}-GFP - *vps39-2* VPS39^{SD}-GFP (NS)	[-1.18, 1.12]
vps39-2 VPS39-RFP – WT (NS)	[-1.1, 0.22]
vps39-2 - *vps39-2* VPS39-RFP (*)	[-2.97, -0.58]

Table S1. Comparison of medians of aborted embryos from Figure 3B. Kruskal-Wallis followed by post-hoc Dunn's test with Šidák correction for multiple comparisons. $p < 0.005116$.

**Genotype Comparison**	**p-value**
WT - *vps39-3* VPS39-GFP	0.23
WT - *vps39-1/+*	$4.82x10^{-13}$
WT - *vps39-1/+* $VPS39^{SA}$-GFP/+:	$2.52x10^{-6}$
WT - *vps39-1/+* $VPS39^{SD}$-GFP/+:	$8.97x10^{-5}$
vps39-3 VPS39-GFP - *vps39-1/+*:	$3.32x10^{-8}$
vps39-3 VPS39-GFP - *vps39-1/+* $VPS39^{SA}$-GFP/+:	$1.50x10^{-3}$
vps39-3 VPS39-GFP- *vps39-1/+* $VPS39^{SD}$-GFP/+:	$1.10x10^{-2}$
vps39-1/+ - *vps39-1/+* $VPS39^{SA}$-GFP/+:	$1.26x10^{-2}$
vps39-1/+ - *vps39-1/+* $VPS39^{SD}$-GFP/+:	$4.74x10^{-3}$
vps39-1/+ $VPS39^{SA}$-GFP/+ - *vps39-1/+* $VPS39^{SD}$-GFP/+:	0.63

Table S2. Pairwise comparison of medians for unfertilized ovules from Figure 3C. Kruskal-Wallis followed by post-hoc Dunn's test with Šidák correction for multiple comparisons. $p < 0.005116$.

**Genotype Comparison**	**p-value**
WT - *vps39-3* VPS39-GFP	$8.199x10^{-2}$
WT - *vps39-1/+*	$1.59x10^{-2}$
WT - *vps39-1/+* $VPS39^{SA}$-GFP/+:	$2.296x10^{-11}$
WT - *vps39-1/+* $VPS39^{SD}$-GFP/+:	$7.80x10^{-4}$
vps39-3 VPS39-GFP - *vps39-1/+*:	0.59
vps39-3 VPS39-GFP - *vps39-1/+* $VPS39^{SA}$-GFP/+:	$7.352x10^{-6}$
vps39-3 VPS39-GFP- *vps39-1/+* $VPS39^{SD}$-GFP/+:	0.13
vps39-1/+ - *vps39-1/+* $VPS39^{SA}$-GFP/+:	$3.67x10^{-5}$
vps39-1/+ - *vps39-1/+* $VPS39^{SD}$-GFP/+:	0.29
vps39-1/+ $VPS39^{SA}$-GFP/+ - *vps39-1/+* $VPS39^{SD}$-GFP/+:	$4.51x10^{-3}$

Table S3. Reference phosphopeptides for PHOT1 and OST1. Guard cell-enriched phosphoproteomics study shows expected phosphorylation in blue light and ABA signaling proteins. Positive Log2FC denotes increased levels of phsphorylation in closed samples. Significant Log2FC > 0.5 or <-0.5. p< 0.05.

Gene symbol	Araport ID	Residue	Log2FC Closed-Open	p-value Closed-Open
PHOT1	AT3G45780	S185	-0.61	$4.43x10^{-5}$
PHOT1	AT3G45780	S350	-0.94	0.01
PHOT1	AT3G45780	S410	-1.86	$6.01x10^{-18}$
PHOT1	AT3G45780	S410	-1.18	$5.59x10^{-7}$
OST1	AT4G33950	S175; T176	2.21	$4.43x10^{-5}$

Table S4. HOPS, CORVET and vacuolar SNARE proteins detected in proteome. Proteins are differentially expressed when Log2FC > 0.5 or <-0.5 and p<0.05.

Gene symbols	Araport ID	AA	Log2FC Closed-Open	p-value Closed-Open
VPS3	AT1G22860	984	0.18	0.15
VPS39	AT4G36630	1000	0.07	0.21
VPS41	AT1G08190	980	0.27	$4.79x10^{-4}$
VPS11	AT2G05170	932	0.27	$3.74x10^{-4}$
VPS18	AT1G12470	988	0.29	$2.59x10^{-4}$
VPS33	AT3G54860	592	0.10	0.23
VTI11	AT5G39510	221	-0.07	0.62
SYP22	AT5G46860	268	-0.14	0.03
SYP51	AT1G16240	232	-0.06	0.32
VAMP713	AT5G11150	221	-0.12	0.13
VAMP727	AT3G54300	240	0.26	$2.83x10^{-3}$

Table S5. Detected phosphorylation of HOPS, CORVET and vacuolar SNARE proteins. Significantly enriched phosphopeptides are those with Log2FC > 0.5 or <-0.5 and p-value <0.05.

Gene symbols	Araport ID	PTM protein localization	Missed cleavages	Log2FC Closed-Open	p-value Closed-Open
VPS39	AT4G36630	S392	0	-0.09	0.56
VPS39	AT4G36630	S413	0	0.79	$5.41x10^{-6}$
VPS39	AT4G36630	S806	0	0.40	$2.102x10^{-3}$
VPS39	AT4G36630	S806	1	-0.08	0.38
VPS41	AT1G08190	S859/S860	2	0.2	0.18
VPS41	AT1G08190	S860	1	-0.02	0.88
VPS41	AT1G08190	S860	2	0.52	0.08
VTI11	AT5G39510	S25	1	-0.1	0.56
VTI11	AT5G39510	S79	2	-0.31	0.06
VAMP727	AT3G54300	S194	0	-0.24	0.07

Table S6. Primers used in this study.

**Primer Name**	**Sequence 5'-3'**	**Purpose**
VPS39-4R	GCTACCGTCTTCTCTAGCACGT	*vps39-2* genotyping
Wisc-DS-Lox-p745	AACGTCCGCAATGTGTTATTAAGTTGTC	*vps39-2* genotyping
VPS39UTR-F	GGTTATTCTCATTCATAGAAGAAAAGCCA	*vps39-2* genotyping
PEX4-q1F	CTT AAC TGC GAC TCA GGG AAT CTT CTA AG	qPCR Housekeeping gene
PEX4-q1R	TCA TCC TTT CTT AGG CAT AGC GGC	qPCR Housekeeping gene
PUX7-qF1	CTCCTCATTCCAAACCCAAAGAGGA	qPCR Housekeeping gene
PUX7-qR1	AAGCAGCTAATGCTCGTTGCAG	qPCR Housekeeping gene
VPS39-Q22F	GGTGGGAGAGGATCAACGGT	qPCR Target gene
VPS39-Q23R	AAACGGAAGCAGGTTATGGAGT	qPCR Target gene
LBb1.3	ATTTTGCCGATTTCGGAAC	*vps39-1* genotyping
VPS39-5R	CACTCTGCCTCAAACTCTTGATGACTG	*vps39-1* genotyping
vps39-1 F1	CTTTGTTGAAGCGTCTTCCAC	*vps39-1* genotyping and phosphomutant genotyping and sequencing
VPS39 Term-R10	CAGACAAACAACACGAAAGGAG	*vps39-1* genotyping and phosphomutant genotyping and sequencing
GFP R1	GTCGTGCTGCTTCATGTGG	phosphomutant genotyping and sequencing
VPS39-RP-GABI	TTGAACTCTCTTTCGCAGCTC	*vps39-3* genotyping
GABI-LB	ATATTGACCATCATACTCATTGC	*vps39-3* genotyping
